# Pharmacological Interaction of Botulinum Neurotoxins with Excitatory and Inhibitory Neurotransmitter Systems Involved in the Modulation of Inflammatory Pain

**DOI:** 10.3390/toxins17080374

**Published:** 2025-07-28

**Authors:** Sara Marinelli, Flaminia Pavone, Siro Luvisetto

**Affiliations:** Institute of Biochemistry and Cellular Biology, National Research Council of Italy, via Ercole Ramarini 32, 00015 Monterotondo, Italy; sara.marinelli@cnr.it (S.M.); flaminia.pavone@cnr.it (F.P.)

**Keywords:** botulinum, inflammatory pain, formalin test, glutamatergic system, GABAergic system, mice

## Abstract

Botulinum neurotoxins (BoNTs) are known to inhibit synaptic transmission by targeting SNARE proteins, but their selectivity toward central excitatory and inhibitory pathways is not yet fully understood. In this study, the interaction of serotypes A (BoNT/A) and B (BoNT/B) with the glutamatergic and GABAergic systems has been investigated using a pharmacological approach in an animal model of inflammatory pain, i.e., the formalin test in mice. BoNTs were administered intracerebroventricularly, three days before testing, followed 15 min before testing by systemic administration of sub-analgesic doses of MK801, an NMDA receptor antagonist, or muscimol, a GABA_A receptor agonist. BoNT/A reduced the second phase of the formalin test without affecting both the first phase and the interphase, suggesting a selective action on excitatory glutamatergic circuits while sparing GABAergic inhibition. Co-administration of MK801 with BoNT/A did not enhance analgesia, and muscimol did not further reduce interphase, confirming preserved GABAergic transmission. In contrast, BoNT/B abolished the interphase, consistent with impaired GABA release. Co-administration of MK801 or muscimol with BoNT/B restored the interphase, indicating compensatory rebalancing of excitatory-inhibitory networks. These results demonstrate that BoNT/A and BoNT/B exert distinct effects on central neurotransmission and support the hypothesis that BoNT/A preferentially targets excitatory synapses, while BoNT/B targets inhibitory synapses. This work contributes to a deeper understanding of anti-inflammatory mechanisms of BoNTs and their selective interaction with central pain pathways.

## 1. Introduction

Botulinum neurotoxin type A (BoNT/A) and B (BoNT/B) are traditionally used in the treatment of dystonia and other motor disorders. Furthermore, BoNT/A has been recognized for its analgesic properties in both clinical and preclinical settings [1,2], and has been suggested as a prophylaxis treatment for several painful conditions, particularly chronic migraine [3]. Its antinociceptive effect relies on the ability to cleave SNAP-25 protein, thereby blocking the release of neurotransmitters such as glutamate, CGRP, and substance P from sensory neurons, particularly those expressing TRPV1 [4,5,6]. Interestingly, BoNT/A appears to exert its effects in a synapse-selective manner, preferentially targeting excitatory glutamatergic neurons while sparing inhibitory GABAergic interneurons [7]. This selectivity may underlie its ability to relieve pain without inducing major side effects related to global synaptic suppression [8].

This hypothesis is supported by behavioural and histological studies showing that BoNT/A spares key inhibitory circuits, including GABAergic interneurons, which are known to play a fundamental role in maintaining the balance between excitation and inhibition in spinal pain pathways. In particular, in the dorsal horn of the spinal cord, GABAergic interneurons regulate the integration and filtering of incoming nociceptive input. Loss or dysfunction of these interneurons, as described in neuropathic conditions, leads to decreased inhibitory tone, contributing to central sensitization and chronic pain [8].

The formalin test represents a powerful experimental model to investigate these mechanisms. A subcutaneous administration of formalin into the animal’s paw, causing an immediate increase in the activity of nociceptive C-afferent fibers, induces a reproducible pattern of nocifensive behaviour characterized by licking, biting, or shaking the injected paw [9]. This behavioural response is typically divided into three phases: an early phase of intense activity of short duration (phase 1; 0–5 min), followed by a phase of relative inactivity (interphase; 5–15 min) which precedes a further phase of activity, longer than phase 1 (phase 2; 15–40 min). Phase 1 involves mainly peripheral mechanisms and is determined by the activation of spinal pathways that transmit nociceptive information to supraspinal centers [10]. The interphase is characterized by a transient suppression of pain behavior, attributed to the activation of an endogenous antinociceptive mechanism, which is sensitive to manipulation of the GABAergic system [11,12,13]. Phase 2 is a prolonged phase of tonic pain reflecting central sensitization, inflammation, and persistent glutamatergic drive at spinal and supraspinal levels [14,15].

The biphasic nature of the behavioral response to formalin injection allows us to investigate whether and how BoNT/A and BoNT/B differentially influence pain transmission, focusing on their interactions with the glutamatergic and GABAergic systems. Previous animal studies have already shown that BoNT/A and BoNT/B elicit different responses in the formalin-evoked inflammatory pain model depending on the route of administration [16,17,18]. In detail, the peripheral administration (subcutaneous; sc) of BoNT/A induced antinociceptive effects during the phase 2 of the formalin test, consistent with attenuation of central glutamatergic signalling, while the sc administration of BoNT/B had no analgesic effect during phase 2 but reduced phase 1 [16]. Even the central administration (intracerebroventricular; icv) of BoNT/A suppressed phase 2, and, more interestingly, both routes of administration did not alter the interphase, suggesting that inhibitory GABAergic tone is preserved. In contrast, icv BoNT/B abolishes the interphase [16], indicating a disruption of GABAergic inhibitory mechanisms likely due to cleavage of VAMP2, which is critical for vesicular release in all neurons, including interneurons. These data suggested a different central modulation of BoNTs on formalin-induced inflammatory pain, and in particular, a different interaction with excitatory and inhibitory neurotransmitter systems, such as the glutamatergic and the GABAergic, which are widely involved in the different phases of the formalin test.

In the present study, to further highlight the mechanism involved in the central modulation of pain, we investigated the pharmacological interaction between centrally administered BoNT/A or BoNT/B and the excitatory and inhibitory systems using sub-analgesic doses of MK801, a non-competitive NMDA receptor antagonist, and muscimol, a GABA_A receptor agonist. Although other models, such as thermal or mechanical hypersensitivity tests, are commonly used, we considered the formalin test the most appropriate behavioural paradigm for the purposes of this study because of its unique biphasic structure that allows for the simultaneous assessment of the excitatory and inhibitory components of pain, making it ideal for the pharmacological analysis of the specific effects of neurotransmitters.

Results of this study demonstrate that the antinociceptive action of BoNT/A in inflammatory pain is tightly linked to suppression of excitatory transmission while sparing endogenous inhibition mediated by GABAergic interneurons. These findings provide important mechanistic insight into BoNT/A’s efficacy and tolerability, with implications for its therapeutic use in pain conditions where preserving spinal inhibitory control is crucial. On the other hand, the lack of antinociceptive action of BoNT/B in inflammatory pain is linked to the suppression of inhibitory transmission mediated by GABAergic interneurons. In light of their potential clinical applicability, the implications of these findings for the selective modulation of central pain pathways will be discussed.

## 2. Results

**Figure 1:**
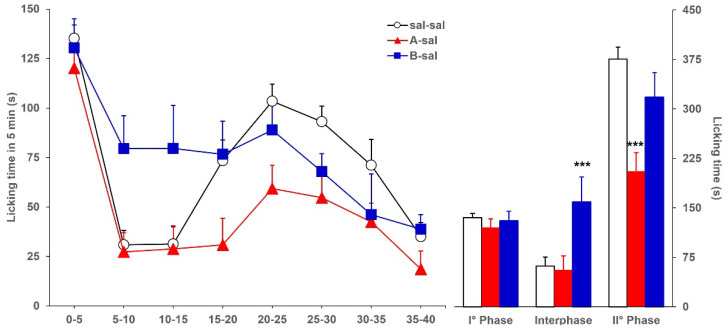
Formalin-induced licking time in sal-sal (◯, *n* = 19), A-sal (▲, *n* = 19), or B-sal (■, *n* = 11) mice. The icv injection of saline, BoNT/A, or BoNT/B was performed 3 days before formalin, while the ip injection of saline was performed 15 min before formalin. Time course of licking duration divided into 5 min periods (left) and cumulative licking time during phase 1 (0–5 min), interphase (5–15 min), and phase 2 (15–45 min) of the formalin test. Statistical significance: (***), *p* < 0.001 vs. sal-sal (Fisher’s PLSD).

As a preliminary experiment, we evaluated whether an intraperitoneal (ip) injection could alter the formalin-induced licking behavior in mice previously icv-injected with saline or BoNTs. Figure 1 shows the licking time, continuously recorded for 40 min and reported as a consecutive 5 min period (left panel) or as cumulative time (right panel) during the three phases of formalin test, evoked by formalin in mice ip-injected with saline and icv-injected with saline (sal-sal) or with 3.75 pg toxin/mouse of BoNT/A (A-sal), or BoNT/B (B-sal). Compared with licking responses in sal-sal (control group) mice, in both A-sal and B-sal mice, the licking time during phase 1 was not significantly altered (*F*_2,46_: 2.015; *p* = 0.1449). Analysis of variance showed a significantly altered licking time during both interphase (*F*_2,46_: 13.199; *p* < 0.0001) and phase 2 (*F*_2,46_: 13.004; *p* < 0.0001). In detail, compared with sal-sal mice, a significant reduction in licking during phase 2 was observed in A-sal mice (*p* < 0.0001), while a significant increase in licking time during interphase was observed in B-sal mice (*p* < 0.0001). No significant alteration of licking time was observed during the interphase in A-sal mice and during phase 2 in B-sal mice. Overall, these results are comparable with those already reported in a previous study [16], in which mice received only one icv injection, without additional ip injection, indicating that a systemic injection *per se* does not alter the licking response of icv-injected mice.

**Figure 2:**
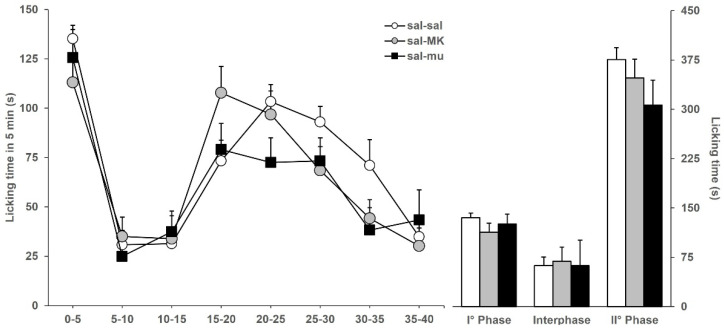
Formalin-induced licking time in sal-sal (◯, *n* = 19), sal-MK (●, *n* = 16), or sal-mu (■, *n* = 11) mice. The icv injection of saline was performed 3 days before formalin, and ip injection of saline, MK801, or muscimol was performed 15 min before formalin. Time course of licking duration divided into 5 min periods (left) and cumulative licking time during phase 1 (0–5 min), interphase (5–15 min), and phase 2 (15–45 min) of the formalin test.

Next, we ensured that the doses of MK801 and muscimol, chosen for the synergistic potentiation experiment, did not significantly alter the licking behavior of saline-injected mice, i.e., they were ineffective and not analgesic *per se*. Figure 2 shows the licking time evoked by formalin in mice (previously icv-injected with saline) ip-injected with saline (sal-sal), or MK801 (sal-MK), or muscimol (sal-mu). Data of sal-sal mice are the same as in Figure 1. Among the three groups of mice considered, no significantly different licking responses were observed, either during phase 1 (*F*_2,46_: 1.840; *p* = 0.1710), interphase (*F*_2,46_: 0.104; *p* = 0.9018), and phase 2 (*F*_2,46_: 1.328; *p* = 0.2757). Results of Figure 2 indicate that the selected doses of MK801 and muscimol, chosen for further analysis of the interaction of BoNTs with the glutamatergic and GABAergic systems, are ineffective and do not induce analgesia.

**Figure 3:**
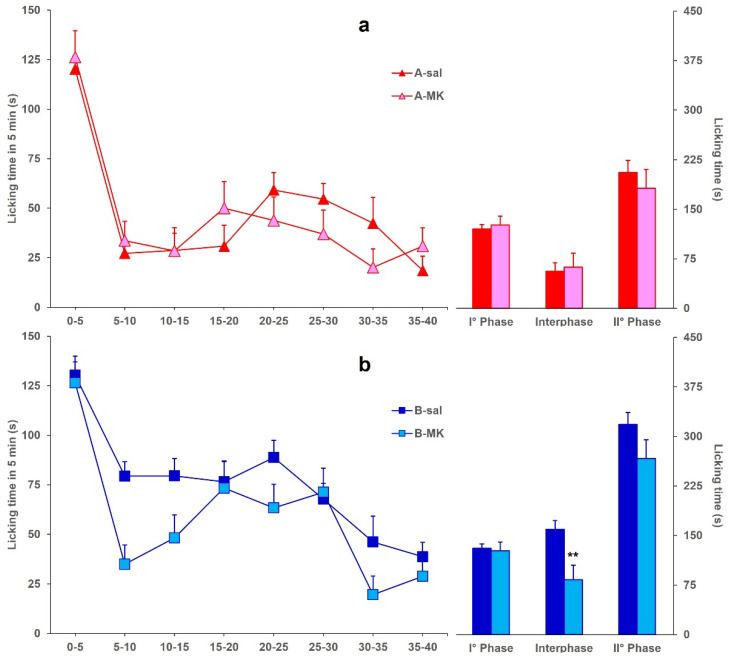
(**a**) Formalin-induced licking time in A-sal (▲, *n* = 19) or A-MK (▲, *n* = 12) mice. The icv injection of BoNT/A was performed 3 days before formalin, and ip injection of saline or MK801 was performed 15 min before formalin. Time course of licking duration (left) and cumulative licking time during the phase 1 (0–5 min), interphase (5–15 min), and phase 2 (15–45 min) of the formalin test. (**b**) Same as in panel A except for BoNT/B (B-sal, ■, *n* = 11; B-MK, ■, *n* = 11) instead of BoNT/A. Statistical significance: (**) *p* < 0.01 vs. B-sal (Fisher’s PLSD).

Figure 3 reports the licking time evoked by formalin in mice icv-injected with BoNT/A (A-MK) or BoNT/B (B-MK) and ip-injected with MK801, compared to A-sal and B-sal mice, considered as control groups, respectively. Data for A-sal and B-sal mice are the same as in Figure 1. Panel (a) shows that the ip injection of MK801 in A-MK mice did not induce a further significant (*F*_1,29_: 0.087; *p* = 0.7704) decrease of licking time in phase 2, with respect to the reduction already induced by BoNT/A. On the other hand, panel (b) shows that ip injection of MK801 restored the interphase in B-MK mice, with a significant difference (*F*_1,20_: 8.141; *p* = 0.0098) between B-sal and B-MK mice.

**Figure 4:**
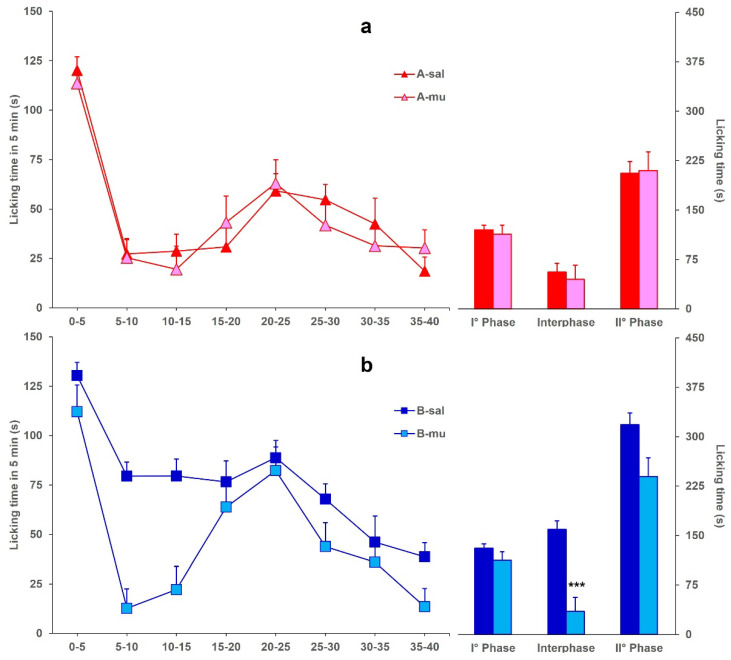
(**a**) Formalin-induced licking time in A-sal (▲, *n* = 19) or A-mu (▲, *n* = 12) mice. The icv injection of BoNT/A was performed 3 days before formalin, and ip injection of saline or muscimol was performed 15 min before formalin. Time course of licking duration (left) and cumulative licking time during the phase 1 (0–5 min), interphase (5–15 min), and phase 2 (15–45 min) of the formalin test. (**b**) Same as in panel A except for BoNT/B (B-sal, ■, *n* = 11; B-mu, ■, *n* = 12) instead of BoNT/A. Statistical significance: (***), *p* < 0.001 vs. B-sal (Fisher’s PLSD).

Finally, Figure 4 shows the licking time evoked by formalin in mice icv-injected with BoNT/A (A-mu) or BoNT/B (B-mu) and ip-injected with muscimol, compared to A-sal and B-sal mice, considered as control groups, respectively. Data for the A-sal and B-sal groups are the same as in Figure 1. Panel (a) shows that the ip injection of muscimol in A-mu mice did not modify the decrease of licking time in phase 2 (*F*_1,29_: 0.356; *p* = 0.5552), with respect to the decrease already induced by BoNT/A (Figure 1). On the other hand, panel (b) shows that ip injection of muscimol in B-mu mice restored the interphase, with a significant (*F*_1,21_: 29.538; *p* < 0.0001) difference between B-sal and B-mu mice.

## 3. Discussion

In line with the hypothesis that BoNTs modulate inflammatory pain by interacting with excitatory and inhibitory neurotransmitter systems, we evaluated the effects of centrally administered BoNT/A and BoNT/B in pharmacological association with drugs affecting glutamatergic or GABAergic systems. The goal was to better understand the mechanisms underlying their differential action and to verify a potential synergistic effect of the combined pharmacological treatment.

**Figure 5:**
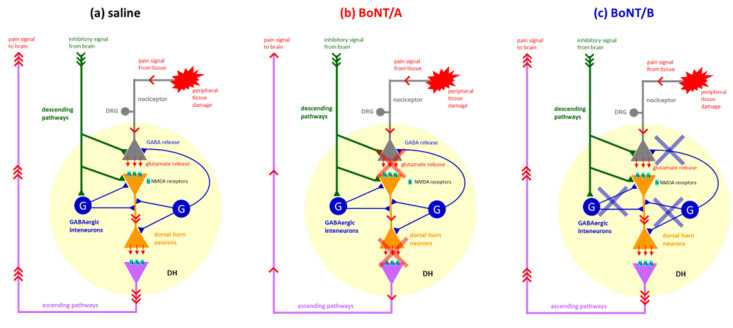
(**a**) Peripheral injection of formalin induces tissue damage that causes an increased activity of primary afferent fibres (gray). In the spinal cord, primary afferents synapse with dorsal horn neurons (orange) where nociceptive information is amplified by excitatory neurotransmission. Pain signals are then transmitted to supraspinal centres via ascending pathways (purple pale). Signals from supraspinal centres activate descending inhibitory pathways (green). Inhibitory substances, such as GABA, serotonin, enkephalins, endorphins, and so on, released from descending pathways act both directly on primary afferents, dorsal horn neurons, and GABAergic interneurons (blue). Activation of GABAergic interneurons induces a further release of GABA that contributes to the quiescent period between the two phases of the formalin test. (**b**) Central administered BoNT/A, 72 h before formalin test, may reach both supraspinal centres and spinal cord dorsal horn, where it reduces the glutamatergic excitatory transmission, thus producing an inhibition of both peripheral and central sensitization with consequent reduction in pain signal transmission (red crosses). The lack of effect of MK-801, an antagonist of postsynaptic NMDA receptors, and muscimol, an agonist of GABA_A receptors, can be explained by considering that, since pain transmission is already reduced by BoNT/A, sub-analgesic doses of MK-801 and muscimol do not further reduce the pain signal. (**c**) Central administration of BoNT/B, by reducing GABA release from both descending pathways and GABAergic interneurons in the dorsal horn (blue crosses), produces a reduction in central inhibitory modulation of the GABAergic system, resulting in loss of the interphase. Restoration of interphase by MK-801 can be explained by considering that GABA levels in the dorsal horn, although reduced by the action of BoNT/B, may be sufficient to balance the residual glutamate release, which in turn is reduced by MK-801. Similarly, the restoration of interphase by muscimol may be a consequence of the enhanced activation of GABA_A receptors, although the GABA levels may be reduced by the action of BoNT/B.

In the first experiment, the NMDA receptor antagonist MK801 was administered at a sub-analgesic dose 15 min prior to the formalin test. Blockade of NMDA receptors can reduce nociceptive responses, particularly during phase 2 of the formalin test [19,20]. However, MK801 did not further reduce phase 2 licking in BoNT/A-pretreated mice, indicating no synergistic effect. The lack of synergistic analgesic effect observed between BoNT/A and MK801 in phase 2 of the formalin test warrants further consideration. A plausible explanation is the occurrence of a ceiling effect: BoNT/A, administered icv 72 h before the test, may have already produced a maximal reduction in central glutamatergic transmission through sustained SNAP-25 cleavage, limiting any additional inhibitory action by MK801. This is consistent with previous findings showing that BoNT/A reduces glutamate release and astrocytic reactivity within 3 days of central administration [21,22]. Another non-mutually exclusive mechanism involves differential receptor saturation. MK801 requires open NMDA channels to exert its non-competitive antagonism; however, BoNT/A-mediated suppression of glutamate release may reduce NMDA receptor activation to subthreshold levels, preventing effective MK801 binding. In such conditions, the NMDA-mediated excitatory drive is already diminished, and further blockade by MK801 becomes functionally redundant. Together, these mechanisms suggest that BoNT/A alone may be sufficient to disrupt central excitatory circuits involved in inflammatory pain processing, thereby occluding the action of additional NMDA receptor antagonists. A hypothetical mechanism of action is depicted in Figure 5.

In contrast, the combination of peripheral MK801 and central BoNT/B administration yielded a paradoxical result: MK801 restored the interphase abolished by BoNT/B [16]. This result implies a compensatory interaction, in which MK801 reduces excessive excitation unmasked by BoNT/B-mediated reduction of GABAergic inhibition, as a consequence of the cleavage of VAMP2 in GABAergic neurons [23]. In addition, GABAergic neurons in the PAG and RVM are critical components of the descending inhibitory system [24,25]. BoNT/B-induced disruption of GABA release in these areas could impair inhibitory tone and explain the abolition of interphase. On the other hand, the interaction with MK801 results in an indirect rebalancing of pain modulation, allowing the behavioral reemergence of the interphase. Similarly, the GABA_A agonist muscimol restored the interphase abolished by BoNT/B. In this context, muscimol binds to and activates GABA_A receptors, enhancing inhibitory chloride currents and thereby increasing neuronal hyperpolarization in postsynaptic neurons [26]. This typically results in the suppression of nociceptive transmission, particularly when GABAergic tone is functionally intact. Data obtained with BoNT/B and muscimol confirm that BoNT/B disrupts GABAergic tone, while muscimol bypasses this presynaptic block and restores inhibitory signaling [11,26]. Conversely, muscimol did not enhance BoNT/A’s effect, likely because excitatory transmission was already suppressed by BoNT/A. This supports earlier evidence that BoNT/A preferentially inhibits glutamate rather than GABA release [27].

Altogether, these data prove that BoNTs effectively block neurotransmitter release in the central nervous system, but they do not affect all synapses equally. BoNT/A preferentially targets excitatory glutamatergic synapses. This is likely due to the higher expression of SNAP-25 in excitatory neurons, which is the specific target of BoNT/A. In addition, another hypothesis suggests that BoNT/A enters specific sensory neurons with high SV2 protein expression, which may explain its selective effects [8,28]. In contrast, BoNT/B, which cleaves VAMP2, disrupted the interphase, leading to an enhanced pain response, suggesting that BoNT/B may directly inhibit GABAergic neurons. According to this hypothesis, muscimol restored the interphase, reversing the disinhibition caused by BoNT/B.

## 4. Conclusions

In conclusion, our study demonstrates that BoNT/A and BoNT/B exert distinct effects on central pain processing, likely due to their differential targeting of glutamatergic and GABAergic neurotransmission. BoNT/A appears to preferentially inhibit excitatory transmission, leading to an effective suppression of phase 2 inflammatory pain responses, while preserving inhibitory tone. In contrast, BoNT/B disrupts GABAergic circuits and abolishes the interphase, a key indicator of intact spinal inhibition, which can be pharmacologically restored by GABA_A or NMDA receptor modulators. These findings suggest that BoNT/A may be the preferred serotype in pain conditions driven by central sensitization and excessive glutamatergic activity, such as inflammatory or neuropathic pain. Conversely, the use of BoNT/B may require careful consideration in contexts where inhibitory networks are already compromised, such as neuropathies involving GABAergic dysfunction. From a translational standpoint, understanding the synaptic selectivity and pharmacodynamic profile of each serotype could help tailor toxin-based treatments to the underlying neurochemical imbalance present in different pain disorders. Further studies are warranted to define serotype-specific indications and refine their clinical application for selective modulation of central pain pathways.

## 5. Materials and Methods

### 5.1. Animals

Four-month-old CD1 male mice (Charles River Labs, Como, Italy), weighing about 35–40 g, were used. Mice were housed in groups of 4 in standard cages under a 12/12 h light/dark cycle (7:00 a.m.–7:00 p.m.), with food and water available ad libitum. Thirty minutes before surgery, mice were transferred to the surgical room and were randomly assigned to treatment groups using a computer-generated randomization list. Each mouse received a unique code unrelated to treatment allocation. Investigators performing behavioral testing and data analysis were blinded to group identity throughout the experimental timeline. The number of mice used is reported in the figure legends. Sample size estimation was performed using G*Power analysis (version 3.1). An a priori analysis was conducted for a repeated-measure ANOVA with a within-between interaction, based on the design of the formalin test (three or two experimental groups x three-time phase: phase 1, interphase, phase 2). All experimental procedures involving animals were conducted in accordance with the guidelines of the Committee for Research and Ethical Issues of the International Association for the Study of Pain (IASP) [29] and complied with the Italian national law (DL116/92), which implemented the European Communities Council Directive 87/609/EEC on the protection of animals used for experimental and other scientific purposes.

### 5.2. Drugs

BoNTs were a kind gift from Prof. C. Montecucco (University of Padova). Activity of BoNTs and concentration of solutions were determined by in vitro cleavage of SNAP-25 (BoNT/A) and VAMP/synaptobrevin (BoNT/B) and the ex vivo mouse hemidiaphragm model. BoNTs were stored at −80 °C in 10 mM NaHEPES, 150 mM NaCl, pH 7.2, and injectable solutions were freshly prepared by dilution of the stock solution in saline (0.9% NaCl). Ketamine, xylazine, MK801, and muscimol were purchased from Sigma-Aldrich (Merck Life Science S.r.l., Milano, Italy).

### 5.3. Surgical Procedure and Drug Injections

To perform icv injections, mice were implanted with a chronic intracranial cannula under anesthesia induced by a mixture of ketamine (100 mg/kg, ip) and xylazine (5 mg/kg, ip) as previously described [30,31]. For icv injection, a volume of 1 μL of saline (0.9% NaCl) or BoNTs solutions was injected 6 days after the surgical implantation of an intracranial cannula with an infusion pump at a rate of 1 μL/min. The dose of BoNTs injected was 3.75 pgtox/mouse, a dose that can be safely injected in mice without toxic effect [31]. The formalin test was performed 3 days after the icv injections, a time we have previously shown to be a necessary time to minimize the possible toxic effects of icv injection of BoNTs [31]. Depending on the experimental group, ip injection of saline, MK801 (0.03125 mg/kg), or muscimol (0.5 mg/kg) was performed 15 min before the formalin test. Sub-analgesic doses of MK-801 and muscimol were chosen based on previous results and from literature [32,33].

### 5.4. Formalin Test

The formalin test was used to measure inflammatory pain as described elsewhere [16]. Formalin solution, 5% in saline, was injected subcutaneously into the dorsal surface of the right hindpaw using a microsyringe equipped with a 26-gauge needle. The licking activity, i.e., the amount of time the mice spent licking, biting, or shaking the injected paw, was automatically recorded for 40 min and calculated in blocks of consecutive 5 min periods. At the end of the recordings, mice were sacrificed to avoid further suffering. Calculation of the behavioral outcome was done after the end of recording.

### 5.5. Experimental Groups

Mice were randomly allocated into nine different groups, based on the combination of icv and ip injections received, as reported in Table 1.

### 5.6. Data Analysis

Data from the formalin test were divided into three phases, phase 1 (0–5 min), interphase (5–15 min) and phase 2 (15–40 min), and analyzed using an ANOVA followed by post hoc comparison conducted using Fisher’s PLSD test. Statistical analysis was performed by using Stat-view software (version 5.1). Differences with *p* < 0.05 were considered significant.

## Figures and Tables

**Table 1:** Experimental mouse groups.

Group Name	icv Injection	ip Injection
sal-sal	saline	saline
A-sal	BoNT/A	saline
B-sal	BoNT/B	saline
sal-MK	saline	MK801
sal-mu	saline	muscimol
A-MK	BoNT/A	MK801
A-mu	BoNT/A	muscimol
B-MK	BoNT/B	MK801
B-mu	BoNT/B	muscimol

## Data Availability

The original contributions presented in this study are included in the article. Further inquiries can be directed to the corresponding author.

## Abbreviations

The following abbreviations are used in this manuscript:

AMPA	α-ammino-3-idrossi-5-metil-4-isossazol-propionic acid
BoNTs	botulinum neurotoxins
BoNT/A, A	botulinum neurotoxin serotype A
BoNT/B, B	botulinum neurotoxin serotype B
GABA	γ-aminobutyric acid
icv	intracerebroventricular
ip	intraperitoneal
MK	MK801
mu	Muscimol
NMDA	N-metil-D-aspartate
sal	saline
sc	subcutaneous
SNAP25	synaptosome associated protein 25
VAMP	vesicle associated membrane protein
